# Phylogenesis of the Functional 1-Aminocyclopropane-1-Carboxylate Oxidase of Fungi and Plants

**DOI:** 10.3390/jof9010055

**Published:** 2022-12-29

**Authors:** Yanan Li, Man Qi, Qi Zhang, Zhixu Xu, Yan Zhang, Yuqian Gao, Yuancheng Qi, Liyou Qiu, Mingdao Wang

**Affiliations:** Key Laboratory of Enzyme Engineering of Agricultural Microbiology, Ministry of Agriculture and Rural Affairs, College of Life Sciences, Henan Agricultural University, Zhengzhou 450002, China

**Keywords:** 1-aminocyclopropane-1-carboxylic acid oxidase, basidiomycete, sequence motif analysis, horizontal gene transfer

## Abstract

The 1-aminocyclopropane-1-carboxylic acid (ACC) pathway that synthesizes ethylene is shared in seed plants, fungi and probably other organisms. However, the evolutionary relationship of the key enzyme ACC oxidase (ACO) in the pathway among organisms remains unknown. Herein, we cloned, expressed and characterized five ACOs from the straw mushroom (*Volvariella volvacea*) and the oyster mushroom (*Pleurotus ostreatus*): VvACO1-4 and PoACO. The five mushroom ACOs and the previously identified AbACO of the button mushroom contained all three conserved residues that bound to Fe(II) in plant ACOs. They also had variable residues that were conserved and bound to ascorbate and bicarbonate in plant ACOs and harbored only 1–2 of the five conserved ACO motifs in plant ACOs. Particularly, VvACO2 and AbACO had only one ACO motif 2. Additionally, VvACO4 shared 44.23% sequence identity with the cyanobacterium *Hapalosiphon* putative functional ACO. Phylogenetic analysis showed that the functional ACOs of monocotyledonous and dicotyledonous plants co-occurred in Type I, Type II and Type III, while putative functional gymnosperm ACOs also appeared in Type III. The putative functional bacterial ACO, functional fungi and slime mold ACOs were clustered in ancestral Type IV. These results indicate that ACO motif 2, ACC and Fe(II) are essential for ACO activity. The ACOs of the other organisms may come from the horizontal transfer of fungal ACOs, which were found ordinarily in basidiomycetes. It is mostly the first case for the horizontal gene transfers from fungi to seed plants. The horizontal transfer of ACOs from fungi to plants probably facilitates the fungal-plant symbioses, plant–land colonization and further evolution to form seeds.

## 1. Introduction

A number of bacteria, fungi, slime molds, algae, and most groups of land plants synthesize, perceive and respond to ethylene, which regulates a variety of physiological activities, including growth, development, reproduction, senescence, and stress responses [1]. However, the ethylene biosynthetic pathways of these organisms are different, including the ethylene-forming enzyme (EFE) pathway, 2-keto-4-methylthiobutyric acid (KMBA) pathway, 1-aminocyclopropane-1-carboxylic acid (ACC) pathway and others. The key enzymes of the ACC pathway are ACC synthase (ACS) and ACC oxidase (ACO). ACS converts methionine-derived S-adenosyl-L-methionine to ACC, which is oxidized to ethylene by ACC oxidase [2,3].

The EFE pathway has been explored for the biosynthesis of ethylene by *Pseudomonas syringae* [4,5,6,7,8], *Ralstonia solanacearum* [9,10] and cyanobacterium *Synechocystis* [11]. Whereas the ethylene biosynthesis pathway in cyanobacterium *Hapalosiphon* is probably the ACC pathway since supplementation with ACC increases ethylene production [12]; however, the ACO of *Hapalosiphon* has not been identified.

Fungal ethylene biosynthesis pathways are quite diverse. The EFE pathway is involved in ethylene production by *Fusarium oxysporum* [13], *Penicillium cyclopium* [14] and *P. digitatum* [15]. The other ascomycetes: *Aspergillus terreus* [16], *Botrytis cinerea* [17,18], and *Fusarium oxysporum* [19], have the KMBA pathway for synthesizing ethylene. Similar to seed plants, the basidiomycetes *Agaricus bisporus* [20,21] and slime mold *Dictyostelium mucoroides* [22] synthesize ethylene via the ACC pathway.

The ethylene biosynthesis pathway in seed plants is a well-known ACC pathway, but the pathway should be absent in non-seed plants [23]. Although several red algae, green algae and mosses can convert ACC to ethylene, such as the red algae *Pterocladiella capillacea* [24], green algae *Haematococcus pluviales* [25], *Acetabularia mediterranea* [26], *Ulva* (*Enteromorpha*) *intestinalis* [27], *Spirogyra pratensis* [28] and moss *Funaria hygrometrica* [29]; however, a liverwort *Riella helicophylla* and fern *Regnellidium diphyllum* [30,31] do not convert [^14^C]-labeled ACC into ethylene. ACO genes are not present in the genomes of species of red algae [32,33], ferns, or bryophytes [34,35] and have not been reported in species of green algae. Thus, the non-seed plants produce ethylene presumably by an unknown non-ACC-dependent pathway [36].

ACO belongs to the 2-oxoglutarate-dependent dioxygenase (2OGD) superfamily of nonheme iron-containing proteins [37]. All 2OGDs have a typical 2-His-1-carboxylate motif required for Fe(II) binding. Dilley et al. [38] proposed the ACO reaction mechanism by using *Malus domestica* ACO1 as a model enzyme. Fe(II) participates in binding to ACC. Several positively charged amino-acid residues from ACO C-terminal α-helix 11, including Arg175, Arg244, Ser246, Lys158, Lys292, Arg299 and Phe300, form a “nest” binding with the other two substrates, ascorbate and bicarbonate. Ascorbate and bicarbonate coordinate the activation the ACO reaction. In particular, ascorbate provides a binding site to ACO for oxygen and donates electrons [38].

The 2OGD superfamily is widely distributed in microorganisms, fungi and mammals and involves many functions [39]; therefore, many proteins have sequence similarities to ACOs. It is difficult to directly extract ACO from biological tissues, and functional characterization of ACO requires the use of recombinant protein. Therefore, only a few functional ACO enzymes have been reported [40]. The phylogenetic analysis of the functional and putative ACOs divides the seed plant ACOs into three types, Type I, Type II, and Type III, and groups non-seed plant ACOs into one cluster of “ancient”, suggesting that the seed plant ACOs diverged from a shared non-seed plant ancestral ACO or 2ODG [40]. Nevertheless, the evolutionary relationship of the functional ACOs between plants and fungi remains unknown.

In previous studies, we found that the compost and casing soil of *Agaricus bisporus* contained ACC [41], and inhibitors that inhibited ACS and ACO in plants also inhibited button mushroom ethylene production. Two ACS genes and one ACO gene were cloned from the *Agaricus bisporus* genome and reduced gene expression decreased ethylene synthesis in *Agaricus bisporus*, similar to plants [42]. Ethylene inhibited mycelial growth and primordium formation but induced post-harvest mushroom maturation and senescence [20,43,44]. In this study, we cloned four and one ACO genes from the straw mushroom (*Volvariella volvacea*) and the oyster mushroom (*Pleurotus ostreatus*), respectively. The heterologously expressed proteins of the five ACO genes showed ACO activities. The evolutionary relationships of the ACOs in fungi and plants were explored.

## 2. Materials and Methods

### 2.1. Strains and Plasmids

*Pleurotus ostreatus* Heikang 650, and *Volvariella volvacea* V23 were provided by Henan Province Edible Fungi Germplasm Resource Bank. *E. coli* BL21, *Pichia pastoris* GS115, pET-28a and pPIC9K were purchased from BioVector NTCC Inc. (Beijing, China).

### 2.2. Bioinformatics Analysis

The ACO protein sequences were aligned using ClustalX 2.0. The phylogenetic tree was constructed by MEGA, version 11.0, using the maximum likelihood method with the bootstrap method (1000 replicates), Poisson model and complete deletion. Motifs were identified by MEME 5.4.1 with the following parameters: “five motifs should MEME find” mode, and other parameters were left as default. An NCBI Conserved Domain Search was used to predict the function of the motifs.

### 2.3. RT-PCR

The mycelia of the straw mushrooms and oyster mushrooms cultured in PDA plates were collected, the total RNA of the mycelia was extracted by the STE method [45], and cDNA was synthesized using HiScript^®^ III RT SuperMix for qPCR (+gDNA wiper) (Vazyme Biotech Co., Ltd., Nanjing, China) following the manufacturers’ instructions. The four ACO genes in the straw mushrooms (designated *VvACO1-4*) and the one ACO gene in the oyster mushrooms (designated *PoACO*) were cloned by PCR. The PCR system contained 100 ng cDNA, 2 μL primer (10 μM), 25 μL 2× Tolo Fast Pfu Premix, and ddH_2_O to reach a total volume of 50 μL. The primers used are listed in Table 1. PCR was performed at 95 °C for 5 min, 32 cycles of 98 °C for 5 s, 57 °C for 30 s, and 72 °C for 30 s, followed by a 72 °C extension for 10 min. The PCR product was purified and then sequenced by Tsingke Biotechnology Co., Ltd. (Zhengzhou, China).

### 2.4. Construction of Mushroom ACO Expression Vectors

The four ACO genes of the straw mushrooms were digested with two enzymes (*Bam*HI, *Hin*dIII, *Eco*RI or *Xba*I) and ligated with the plasmid pCold TF or pET-28a to obtain prokaryotic expression vectors. The ACO gene of the oyster mushrooms was digested with *Eco*RI and *Not*I and then ligated with the plasmid pPIC9K to obtain a eukaryotic expression vector.

### 2.5. Mushroom ACO Expression and Purification

Four ACO prokaryotic expression vectors of the straw mushrooms were transformed into *E. coli* BL21, which was cultured in LB medium at 37 °C and 220 rpm to an OD_600_ of 0.4 and then induced by 0.5 mM IPTG at 16 °C and 180 rpm for 12 h. The target proteins were purified using a HisTrap^TM^ HP gravity flow column (GE Healthcare, Uppsala, Sweden), eluted with 200 mM–250 mM imidazole, and the eluate was detected by SDS-PAGE.

The oyster mushroom ACO eukaryotic expression vector was transformed into *Pichia pastoris* GS115 using the electroporation method. The transformants were grown and induced to express proteins according to methods described elsewhere [46]. The culture supernatant was collected and concentrated in a 10 kDa ultrafiltration tube. The concentrated solution was determined by SDS-PAGE.

### 2.6. ACO Enzyme Activity Assay

The enzyme activities of the expressed ACO proteins were assayed using a previously described method [21] with a slight modification. Briefly: a 5.5 mL headspace vial contained a 1.8 mL mixture composed of 150 mM NaHCO_3_, 30 mM ascorbic acid, 1.0 mM ACC, 0.1 mM FeSO_4_, and 0.1 mg enzyme protein in 100 mM Tris-HCl buffer at pH 7.2. After shaking incubation at 30 °C for 1 h, 1 mL gas was withdrawn for ethylene determination by gas chromatography (GC-2010 plus, Shimadzu, Kyoto, Japan) [47].

## 3. Results

### 3.1. Cloning, Expression and Enzyme Activities of ACO Proteins from Straw Mushrooms and Oyster Mushrooms

There are four ACO genes annotated in the straw mushroom *V. volvacea* V23 genome [48] and designated *VvACO*1-4 (Table 2). One ACO gene was annotated in the oyster mushroom *P. ostreatus* PC15 genome [49] and named *PoACO* (Table 2). The primers were designed according to the ACO gene sequences (Table 1), and four and one ACO genes were cloned from the mRNA of the straw mushroom *V. volvacea* V23 and *P. ostreatus* Heikang 650, respectively. After sequencing and alignment, the sequences of the five genes were completely consistent with the genome data.

**Figure 1:**
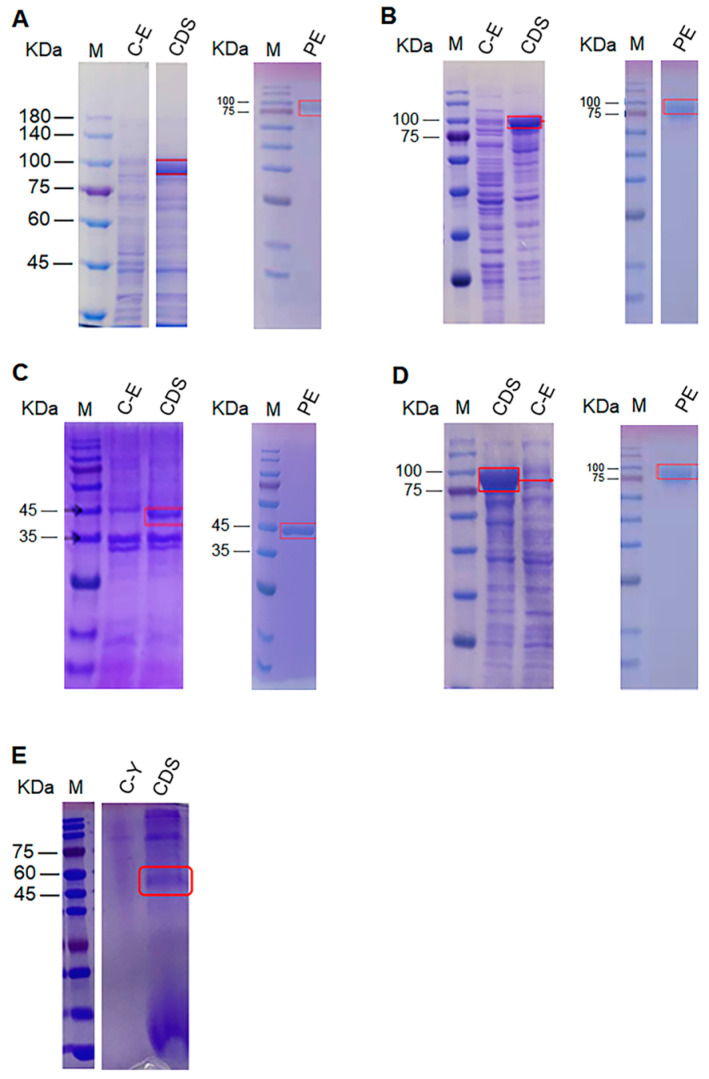
Expression and purification of the ACO proteins from straw mushrooms and oyster mushrooms. (**A**), VvACO1; (**B**), VvACO2; (**C**), VvACO3; (**D**), VvACO4; (**E**), PoACO. M, protein marker; C-E, control *E. coli* cell extract; CDS, cell disruption supernatant; C-Y, control yeast cell extract; PE, purified enzyme.

The predicted molecular weights of VvACO1-4 were 39.97 kD, 42.08 kD, 42.04 kD and 39.44 kD, respectively. We first used the pET-28a plasmid to express the four proteins, but only VvACO3 was successfully expressed (Figure 1C). The other three proteins were expressed using the pCold TF plasmid. The molecular weights of VvACO1, VvACO2 and VvACO4 expressed by pCold TF vectors were approximately 90, 92, and 90 kDa, respectively, due to the molecular weight of TF (trigger factor from *E. coli*) being approximately 50 kDa (Figure 1A,B,D).

PoACO protein had an expected molecular weight of 36.95 kDa. We used pET-28a and pCold TF vectors to express PoACO but could not obtain free active protein. Then, we successfully obtained free active protein using the plasmid pPIC9K expressed in *Pichia* yeast. The molecular weight of the PoACO protein was approximately 50 kDa (Figure 1E), probably caused by glycosylation modification.

**Figure 2:**
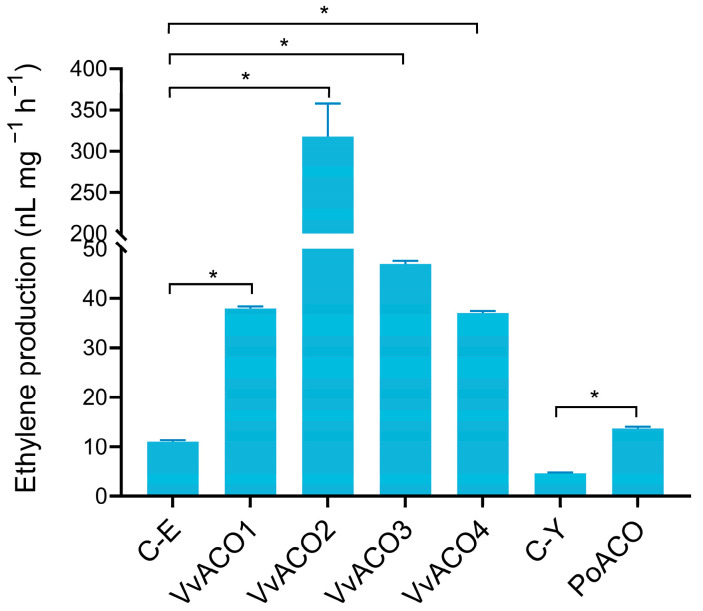
Specific activities of the heterologously expressed ACO enzymes from straw mushroom and oyster mushroom. The data shown were the mean of three independent experiments ± standard deviation (SD). They were analyzed by one-way analysis of variance (ANOVA) followed by the Tukey–Kramer multiple-comparison post hoc test. * Indicates a significant difference at *p* < 0.05.

The ability of the purified proteins to oxidize ACC to produce ethylene was determined. All five proteins were able to oxidize ACC to produce ethylene, among which VvACO2 had the highest specific enzyme activity (Figure 2).

### 3.2. Residue Analysis of Functional Fungi, Slime Mold and Plant ACOs

**Figure 3:**
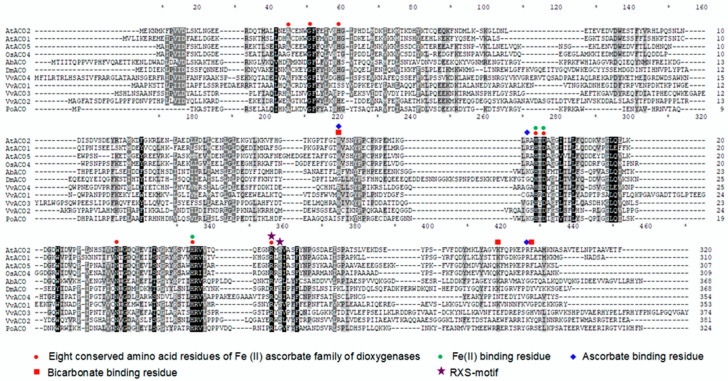
Selected functional ACO protein sequence alignment of mushrooms, slime molds and plants. Protein sequences were aligned using ClustalX 2.0. The important and conserved amino acid residues were marked in accordance with the legend shown.

Fungal functional ACOs included AbACO from button mushrooms [20,21], VvACO1-4 from straw mushrooms and PoACO from oyster mushrooms identified in this study. The functional ACO of slime mold was DmACO from *D. mucoroides* [22]. These seven functional ACOs were sequence aligned with the four representative plant-functional ACOs AtACO2 (Type I), AtACO1 (Type II), and AtACO5 and OsACO4 (Type III). Except for the sequence identity of PoACO and AbACO, which reached 41.36%, the sequence identity among the ACO proteins of other fungi and slime molds was 19.4–31.3%. The ACO proteins of these fungi and slime molds were only 18.0–27.3% identical in sequence to those of plants. Six of the eight conserved amino acid residues of the Fe(II) ascorbate family of dioxygenases [21] were harboured in all 11 ACO proteins, and the other two residues were variable. The two variable residues can occur in both plant ACOs, five fungal ACOs and slime mold ACOs. The three conserved Fe(II) binding residues in plant ACOs [64] were all involved in the six conserved residues, whereas the proposed ascorbate binding residues [38] and bicarbonate binding residues [65] in plant ACOs were all highly variable in fungal and slime mold ACOs. In addition, an entirely conserved RXS motif proposed to be involved in binding the carboxylate of ACC in plant ACOs [66] was also variable in XS in fungal ACOs (Figure 3).

### 3.3. Phylogenetic Analysis of Microbial and Plant ACOs

**Figure 4:**
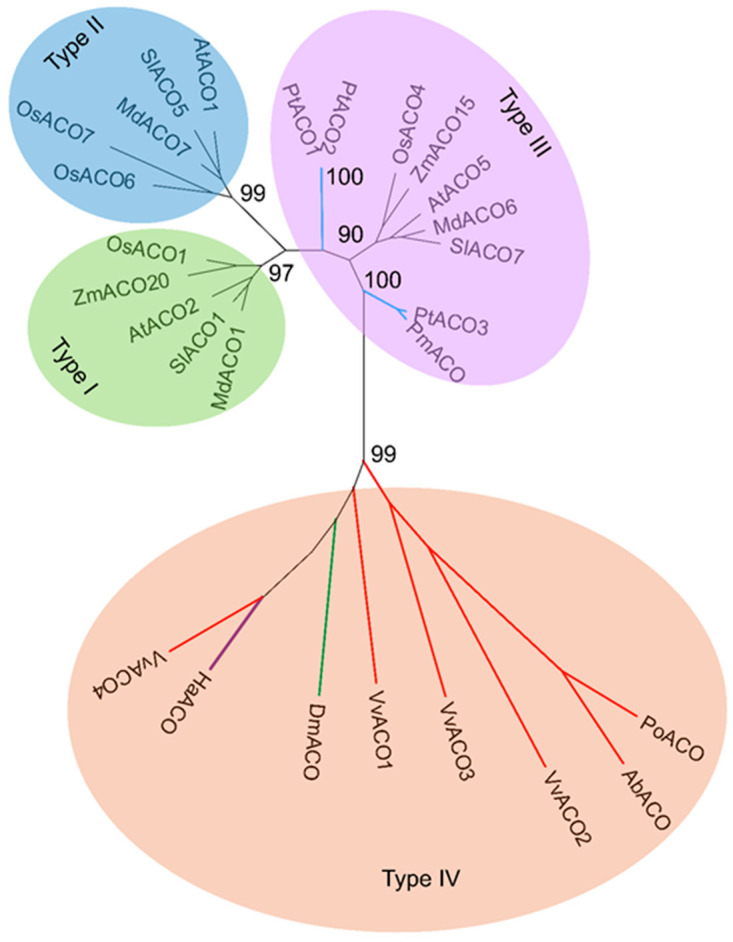
Unrooted phylogenetic tree of 27 ACO proteins generated by MEGA, version 11.0, with the maximum likelihood method. Numbers near branches show percentage bootstrap support. Red clades indicate fungal ACO proteins; blue clades indicate ACO proteins of gymnosperms; green clades indicate the ACO proteins of slime mold; purple clades indicate bacterial ACO proteins; black clades indicate the ACO proteins of angiosperms. The protein sequence information is listed in Table 2.

The identified functional ACOs of fungi, slime molds and some angiosperms and possibly functional ACOs of several bacteria and gymnosperms (Table 2) were selected to perform phylogenetic analysis to study their evolutionary relationships. These 27 ACOs should be divided into four types. Type I, Type II and Type III all contained the ACOs of monocotyledonous and dicotyledonous plants, while ACOs of gymnosperms were all grouped into Type III. Type IV contained only the ACOs of fungi, slime molds and bacteria (Figure 4).

**Figure 5:**
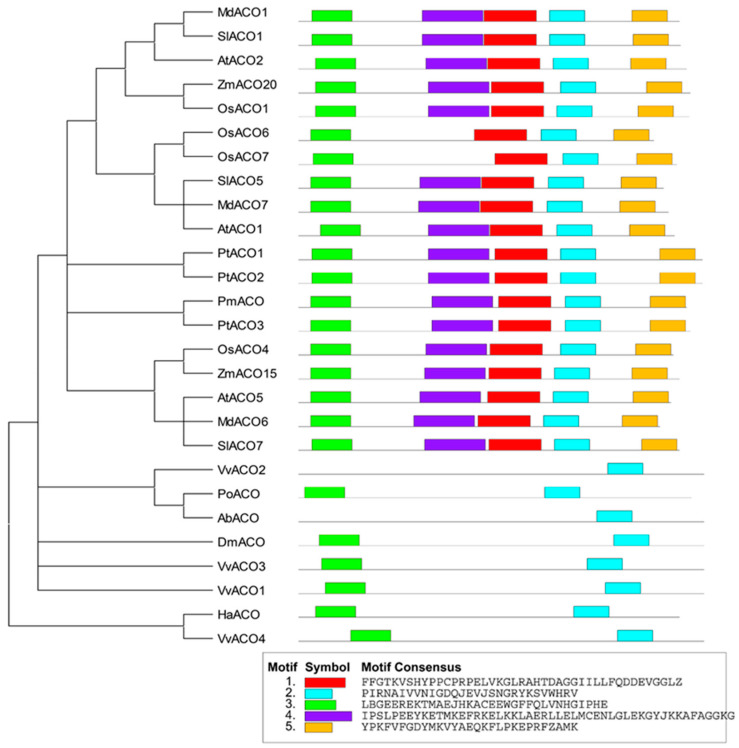
The phylogenetic relationships and conserved motif structure of the ACO protein sequences from the functional ACOs of fungi, slime molds and angiosperms plants, and the putative functional ACOs of cyanobacteria and gymnosperms. The phylogenetic trees were constructed MEGA version 11.0 with the maximum likelihood method. The conserved motif structure was discovered by MEME. The protein sequence information listed in Table 2.

Five conserved motifs were discovered in the 27 ACOs, which all belong to ACO motifs. The seed plant ACOs all contained these five conserved motifs, except OsACO6 and OsACO7, which had four conserved motifs. The ACOs of bacteria, fungi and slime moulds had only 1–2 conserved motifs, especially VvACO2, and AbACO had only one ACO motif 2 (PIRNAIVVNIGDQJEVJSNGRYKSVWHRV) (Figure 5).

## 4. Discussion

Five putative ACO genes from straw mushrooms and oyster mushrooms were cloned and expressed, and all showed ACO activities, such as the button mushroom AbACO and slime mold DmACO. The amino acid sequence identity between AbACO and plant ACOs is less than 25%, and the conserved ascorbate binding residues, bicarbonate binding residues and RXS in plant ACOs are not conserved in AbACO. The three conserved residues that bind Fe(II) in plant ACOs are conserved in AbACO. Mutation of the three conserved residues that bind Fe(II) reduces the specific activity of AbACO by 24–38%. However, mutating the G in the RXG of AbACO to S increased the specific activity of the enzyme two-fold and dramatically reduced the bicarbonate dependence. It is suggested that AbACO activity requires ACC, Fe(II) and bicarbonate but does not require ascorbate [21]. Similar to AbACO, 2–3 residues out of the three conserved and binding ascorbate residues in plant ACOs were not conserved in fungi and slime molds. Of the three conserved residues that bind bicarbonate in plant ACOs, one and 2–3 were not conserved in AtACO1 and the ACOs of fungi and slime molds. The RXS motif conserved in plant ACOs was replaced with RXG in PoACO, as in AbACO. The ACOs of straw mushroom, oyster mushroom and slime mold had 1–2 conserved ACO motifs, whereas the ACOs of plants had 4–5. In particular, VvACO2 and AbACO had only one ACO motif 2, indicating that ACO motif 2, ACC and Fe(II) should be essentially required for ACO activity.

Despite the fact that several ethylene receptors and ethylene signal transduction components of seed plants may originate from green algae [28], the ACOs in seed plants should not come from green algae. Houben and Van de Poel conducted a phylogenetic tree analysis of the ACOs of angiosperm plants along with putative ACOs of gymnosperms and other non-seed plants (without fungi), dividing these ACOs into Type I, Type II, Type III and the other group of the ancestral non-seed plant node of putative ACOs. Type I and Type II include ACOs of common angiosperm plants, and Type III includes ACOs of common angiosperm plants and putative ACOs of some gymnosperms. They proposed that the three types of ACOs diverged in parallel from a shared non-seed plant ancestral ACO [40]. However, neither putative *ACO* genes from gymnosperms nor non-seed plants were functionally characterized in vitro. The putative ACOs of the gymnosperms and other non-seed plants were almost all not ACOs but other 2OGDs.

The putative ACO protein PmACO of *Pseudotsuga menziesii* was able to bind to the *Arabidopsis* ACO antibody. Its expression was upregulated by mechanical wounding, consistent with the wound-induced increase in ethylene levels [62]. Three putative ACO genes, *PtACO*1-3, were cloned from *Pinus taeda*, and their promoters responded to indole-3-acetic acid (IAA), wounding, and gravitropic reorientation [63,64]. Cyanobacterium *Hapalosiphon* may have the ACC pathway to synthesize ethylene [12]. Two isopenicillin N synthase family oxygenases were annotated in the genome of *Hapalosiphon* sp. MRB220. One of them was 44.23% identical to VvACO4 in the deduced amino acid sequence and very likely to be ACO, named HaACO (GenBank WP_053457617.1). In this study, phylogenetic analysis of the identified functional ACOs of fungi, slime molds and some angiosperms, and possibly functional ACOs of several bacteria and gymnosperms, showed that the ACOs can be divided into four types. The ACOs of seed plants were grouped into Type I, Type II and Type III, similar to the above report [40]. Nevertheless, the ancestral Type IV contained only fungi, slime molds and bacteria. This suggests that the ACOs of seed plants may originate from microorganisms.

This study and a previous report [40] showed that the ACOs of monocotyledonous and dicotyledonous plants co-occur in Type I, Type II and Type III. Gymnosperm ACOs also appeared in Type III, implying the existence of horizontal gene transfer of ACOs in seed plants. In this study, the ACOs of bacteria, fungi and slime molds were clustered in Type IV, indicating that there was also horizontal gene transfer among them. ACOs are rarely found in bacteria but ordinarily in basidiomycetes, and the sequence identity of cyanobacterial HaACO and VvACO4 is as high as 44.23%, indicating that HaACO is likely to be horizontally transferred from fungi. Fungal ACOs contained only 1–2 conserved ACO motifs and were quite primitive and simple compared to plant ACOs. In addition, ACOs were not found in non-seed plants [34]. Therefore, ACOs in seed plants should be horizontally transferred from fungi. Horizontal gene transfers from fungi to seed plants have not been discovered previously [66]. The horizontal gene transfers from fungi to seed plants found in this study may be the first to be reported.

Ascomycota and Basidiomycota diverged about 500 million years ago—a little time before plants started their colonization of land [67]. The horizontal transfer of ACOs from fungi to plants probably facilitates fungal–plant symbioses, plant–land colonization and further evolution to form seeds.

## 5. Conclusions

We cloned, expressed and characterized five ACOs from basidiomycetes straw mushrooms and oyster mushrooms. The five ACOs, plus the previously identified AbACO of button mushroom, contained all three conserved residues that bind to Fe(II) in plant ACOs but did not contain the entire residues that conserved binding to ascorbate and bicarbonate in plant ACOs and only had 1–2 of the five conserved ACO motifs in plant ACOs. Interestingly, VvACO4 shared 44.23% sequence identity with the putative cyanobacterial HaACO. Evolutionary analysis of the functional ACOs of fungi, slime molds and angiosperm plants and the putative functional ACOs of cyanobacteria and gymnosperms can classify these ACOs into four types. The ACOs of monocotyledonous and dicotyledonous plants cooccurred in Type I, Type II and Type III. Gymnosperm ACOs also appeared in Type III. The ACOs of bacteria, fungi, and slime molds were clustered in Type IV. These results indicate that ACO motif 2, ACC and Fe(II) are essential for ACO activity. Horizontal gene transfer of ACOs exists between monocotyledonous and dicotyledonous plants, between seed plants and gymnosperms and between fungi and bacteria. The ACO genes of the other organisms may come from the horizontal transfer of fungal ACO genes.

## Figures and Tables

**Table 1:** List of primers used in the present study.

Name	Nucleotide Sequence (5′–3′)	Application
PoACO-F	ATGCCTACGAAGGCATCTACA	Cloning of PoACO
PoACO-R	TTAGTTGAAATGCTTAATAACAGTTCCACGAATCC	
pPIC9K-ACO-F	AAGGCGAATTAATTCGCGGCCGCATGCCGACCAAAGCAAGCA	Construction for pPIC9K-PoACO
pPIC9K-ACO-R	GCTGAAGCTTACGTAGAATTCTTAATGATGATGATGATGATGAA- TCACTGTACCACG	
VvACO3-F	GGATCCATGTCCCATCTCAATTCTGCAGCC	Cloning of VvACOs and Construction for the VvACOs expression vectors
VvACO3-R	AAGCTTTTAATAGGCACCGACTTGCCCG	
VvACO1-F	GGAATTCCATATGATGGCGGCTCCTAAATCTACC	
VvACO1-R	GCTCTAGATTAATACCTCCCCTTCTTCTCGTC	
VvACO2-F	CGGGATCCATGGCTGGCTTTGCCACGT	
VvACO2-R	GCTCTAGATCAAGCCTCAATGCGCTCAG	
VvACO4-F	CGGGATCCATGTTTATCTTAAGAACTCGGCTTC	
VvACO4-R	GCTCTAGATTACGAGTAGGTCACTGCCAGG	

**Table 2:** Selected ACO sequences used in the phylogenetic analyses.

Species	Protein Name	Protein ID	Type	Protein (aa)	Reference
*Volvariella volvacea*	VvACO4	JGI 111930	4	354	This study
	VvACO3	JGI 116615	4	374	This study
	VvACO2	JGI 118606	4	381	This study
	VvACO1	JGI 111142	4	353	This study
*Pleurotus ostreatus*	PoACO	KDQ32580	4	324	This study
*Agaricus bisporus*	AbACO	JGI 195789	4	368	[20]
*Dictyostelium mucoroides*	DmACO	BAF64840	4	368	[22]
*Arabidopsis thaliana*	AtACO1	AT2G19590.1	2	310	[50]
	AtACO2	AT1G62380.1	1	320	[51]
	AtACO5	AT1G77330.1	3	307	[50]
Apple (*Malus domestica*)	MdACO1	MDP0000195885	1	314	[52]
	MdACO6	MDP0000025650	3	298	[53]
	MdACO7	MDP0000200896	2	305	[54]
Tomato (*Solanum lycopersicum*)	SlACO1	Solyc07g049530.2.1	1	315	[55]
	SlACO5	Solyc07g026650.2.1	2	301	[56]
	SlACO7	Solyc06g060070.2.1	3	314	[57]
Maize (*Zea mays*)	ZmACO20	Zm00008a017510_T01	1	323	[58]
	ZmACO15	Zm00008a037502_T01	3	314	[22]
Rice (*Oryza sativa*)	OsACO1	LOC_Os09g27820.1	1	322	[59]
	OsACO6	LOC_Os06g37590.1	2	293	[60]
	OsACO7	LOC_Os01g39860.1	2	312	[22]
	OsACO4	LOC_Os11g08380.1	3	309	[22]
Douglas fir (*Pseudotsuga menziesii*)	PmACO	ABF20554	4	320	[61]
Loblolly pine (*Pinus taeda*)	PtACO1	ADD65762	4	333	[62,63]
	PtACO2	ADD65761	4	333	[62,63]
	PtACO3	ADD65760	4	323	[62,63]

## Data Availability

Not applicable.
